# Hyperjaponol H, A New Bioactive Filicinic Acid-Based Meroterpenoid from *Hypericum japonicum* Thunb. ex Murray

**DOI:** 10.3390/molecules23030683

**Published:** 2018-03-18

**Authors:** Rongrong Wu, Zijun Le, Zhenzhen Wang, Shuying Tian, Yongbo Xue, Yong Chen, Linzhen Hu, Yonghui Zhang

**Affiliations:** 1 National & Local Joint Engineering Research Center of High-throughput Drug Screening Technology, Hubei Key Laboratory of Biotechnology of Chinese Traditional Medicine, School of Life Science, Hubei University, Wuhan 430062, China; wrr08291798@163.com (R.W.); TSY15008202259@163.com (S.T.); yongchen101610@163.com (Y.C.); 2 Wuhan Rayson School, Wuhan 430040, China; zijunle2018@163.com; 3 Hubei Key Laboratory of Natural Medicinal Chemistry and Resource Evaluation, School of Pharmacy, Tongji Medical College, Huazhong University of Science and Technology, Wuhan 430030, China; wzz75283@163.com (Z.W.); yongboxue@hust.edu.cn (Y.X.); zhangyh@mails.tjmu.edu.cn (Y.Z.)

**Keywords:** *Hypericum japonicum*, meroterpenoid, Epstein-Barr virus

## Abstract

Hyperjaponol H (**1**), a new filicinic acid-based meroterpenoid, with a 6/6/10 ring system *trans*-fused by hetero-Diels–Alder cycloaddition between a germacrane sesquiterpenoid and a filicinic acid moiety, was isolated from aerial parts of *Hypericum japonicum*. The elucidation of its structure and absolute configuration were accomplished by the analyses of extensive spectroscopic data and the comparison of Cotton effects of electron circular dichroism (ECD) with previously reported ones. The bioactivity assay showed that hyperjaponol H exhibited a moderate inhibitory efficacy on lytic Epstein-Barr virus (EBV) DNA replication in B95-8 cells.

## 1. Introduction

Natural products are widely known to be a considerable resource of biologically active compounds that involve manifold and unusual scaffolds. Most secondary metabolites from plants of Guttiferae are mainly found to be phloroglucinol derivatives with complex architectures and appealing therapeutical properties [1,2,3]. *Hypericum japonicum* Thunb. ex Murray, as a member of Guttiferae family, also termed as Tianjihuang in China, is widespread chiefly in temperate regions of North America, Oceania, and Asia [4,5]. Also as a type of traditional Chinese medicine, *H. japonicum* has been extensively utilized for the medical treatment of the hemostasis, detumescence, dysentery, and hepatitis [4]. In recent years, literatures reported that various ranges of chemical constituents such as aliphatic compounds, terpenoids, flavonoids, xanthonoids, lactones, and phloroglucinol derivatives had been discovered from this herb [5,6,7,8,9,10,11].

In our on-going research on the genus *Hypericum* for structurally fascinating and biologically appealing metabolites, we have reported some meroterpenoids of polycyclic prenylated acylphloroglucinols (PPAPs) from *H. sampsonii*, *H. ascyron*, *H. attenuatum*, and *H. perforatum* [12,13,14,15] as well as a series of filicinic acid-based meroterpenoids (Hyperjaponols A–G) from *H. japonicum* [16]. In the present study, the isolation, structural confirmation, and anti-EBV assay of compound **1**, named hyperjaponol H, a metabolite of *H. japonicum*, are illustrated in detail.

## 2. Results and Discussion

**Figure 1:**
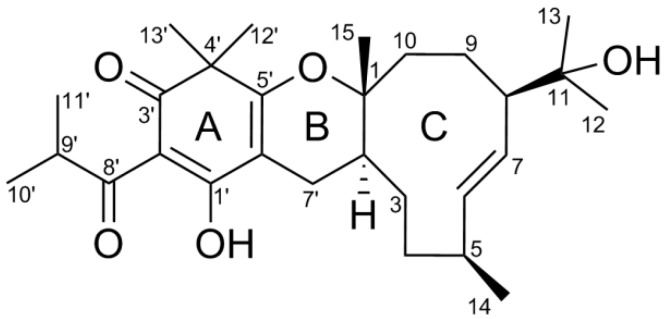
Structure of compound **1**.

A crude extract (300 g) produced from the dried herbs of *H. japonicum* (4 kg) was subjected to the silica gel column chromatography (silica gel CC) eluted successively with the gradient mobile phases of petroleum ether, chloroform, and ethyl acetate. The fraction of petroleum ether was sequentially chromatographed by MCI gel column, ODS Middle Pressure Liquid Chromatography (MPLC), Sephadex LH-20, and High Performance Liquid Chromatography (HPLC) to give a new filicinic acid-based meroterpenoid (**1**) as drawn in Figure 1, which was named as hyperjaponol H.

Hyperjaponol H (**1**), white amorphous powder {[α]${}_{D}^{20}$ +16.4 (*c* 0.06, CHCl_3_)}, with the molecular formula C_28_H_42_O_5_, was manifested by the high-resolution electrospray ionization mass spectra (HRESIMS) quasi-molecular peak ion at *m*/*z* 459.3119 [M + H]^+^, calculated for C_28_H_43_O_5_ 459.3110 (Supplementary Materials Figure S1). The analyses of IR absorption bands (3455, 1654, and 1612 cm^−1^, Supplementary Materials Figure S9) demonstrated the characteristic scaffold of an enolic 1,3-diketo system, viz. acylated filicinic acid parent core [10,11,17,18]. Comparison of NMR data between **1** with hyperjaponols A–G [16] indicated that **1** was constructed via the incorporation of the same sesquiterpene unit as hyperjaponol G to the same acylated filicinic acid entity as hyperjaponols A–F. Accomplished by meticulous examination of HSQC, HMBC, and ^1^H-^1^H COSY spectra (Supplementary Materials Figures S4–S6), all ^1^H- and ^13^C-NMR data of **1** were unequivocally assigned as shown in Table 1. The ^1^H-NMR (600 MHz) spectrum (Supplementary Materials Figure S2) displayed resonances for eight methyls [δ_H_, 1.29 (s), 1.24 (s), 1.20 (s), 1.19 (s), 1.02 (s), 1.11 (*d*, *J* = 6.7 Hz), 1.10 (*d*, *J* = 6.7 Hz), 1.00 (*d*, *J* = 7.0 Hz)], and two olefinic methine protons [δ_H_, 5.59 (*dd*, *J* = 16.2, 4.7 Hz), 5.47 (*dd*, *J* = 16.2, 8.0 Hz)]. The ^13^C-NMR (150 MHz) spectrum (Supplementary Materials Figure S3) denoted 28 carbon resonances consisting of one quaternary carbon, one carbonyl, two oxygenated, and five enolic or olefinic carbons, five methylenes, six methines (four aliphatic, and two olefinic carbons), and eight methyls. Taking the aforementioned analyses and its eight indices of proton deficiency into consideration, compound **1** contains a tricyclic system.

**Figure 2:**
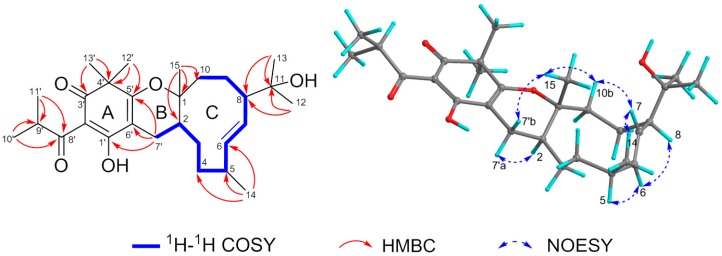
Key 2D NMR correlations of compound **1**.

The planar construction of **1** was established according to the HMBC and ^1^H-^1^H COSY experiments (Figure 2). In ring C, the 2-hydroxyisoisopropyl residue was connected at position 8 due to the HMBC correlations from Me-12/Me-13 to C-11 and C-8, while the HMBC cross-peaks between Me-15 with C-1, C-2, and C-10 as well as the cross-peaks between Me-14 with C-4, C-5, and C-6 suggested Me-15 and Me-14 was located at positions 1 and 5, respectively. Meanwhile, the clear ^1^H-^1^H COSY spin systems of H-2/H-3/H-4/H-5/H-6/H-7/H-8/H-9/H-10 supported the structural profile of ring C, a germacrane unit. Regarding to the ring A, a filicinic acid core, was confirmed by the HMBC correlations of Me-12′/Me-13′ with C-3′, C-4′, and C-5′, H-7′ with C-1′, C-5′, and C-6′ along with an unassigned olefinic carbon (δ_C_ 104.6) referring to literatures [10,11,17,18]. In addition, the isobutyryl functionality positioned at C-2′ was illustrated by HMBC correlations from Me-10′/Me-11′ to C-8′ and C-9′. Definitively, the combination of the filicinic acid (ring A) and the germacrane (ring C) via C-7′ was established by the ^1^H-^1^H COSY spin system of H-2/H-7′, and ring B formed to fit the unsaturation degrees of **1**.

According to the 2D NOESY spectrum (Supplementary Materials Figure S7) and ^1^H-^1^H coupling constant, the relative configurations of the chiral centers of **1** were revealed. In light of a large coupling constant value of H-6/H-7 (*J* = 16.2 Hz), an E geometry of the olefinic bond (∆^6,7^) was ascertained. NOE correlations between Me-14/H-7, H-7/H-10b (δ_H_ 2.03), H-10b/Me-15, and Me-15/H-7′b (δ_H_ 1.69) suggested that these protons should be assigned as the same side named β orientation. Analogously, the observed NOE cross-peaks between H-7′a (δ_H_ 2.74)/H-2, H-6/H-5, and H-6/H-8 as well as the absence of a key NOESY correlation between H-2/Me-15, indicated H-2, H-5, H-6, and H-8 should be placed at α orientation (Figure 2). Furthermore, the value of ^3^*J*_H-7′b__−__H-2_ (*J* 11.3 Hz) suggested that a dihedral angle 180 between H-7′b and H-2 assigned these two protons as trans-stereochemistry. Thus, a 6/6/10 ring system was incorporated by the sesquiterpenoid germacrane entity trans-fused into the acylfilicinic acid motif, which possessed a 1R*,2S*,5S*,8R* relative configuration.

**Figure 3:**
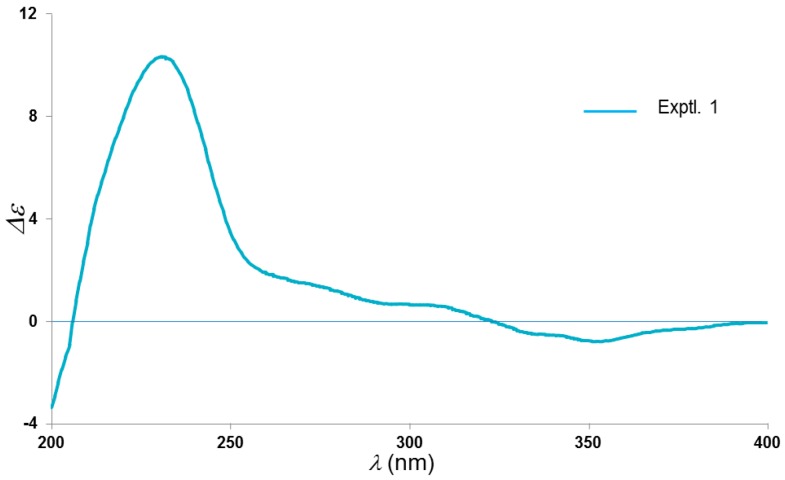
Experimental ECD spectrum of **1** (in CH_3_OH).

As confirmation, the absolute stereocenters of C-1, C-2, C-5, and C-8 in **1** were assigned by means of the cautious comparison of electronic circular dichroism (ECD) data between **1** and its homologues, i.e., hyperjaponols D–G, with the identical sesquiterpenoid germacrane. The ECD spectra exhibited positive Cotton effects at 226–231 nm (ECD (CH_3_OH) λ (*Δε*): **1**, 231 (+10.34) (Figure 3); hyperjaponol D, 227 (+3.23); hyperjaponol E, 227 (+5.28); hyperjaponol F, 229 (+4.48); hyperjaponol G, 226 (+13.74)] together with the dextrorotatory optical activities of compound **1** and hyperjaponols D–G, which designated the stereochemistry of **1** as 1*R*,2*S*,5*S*,8*R*.

**Figure 4:**
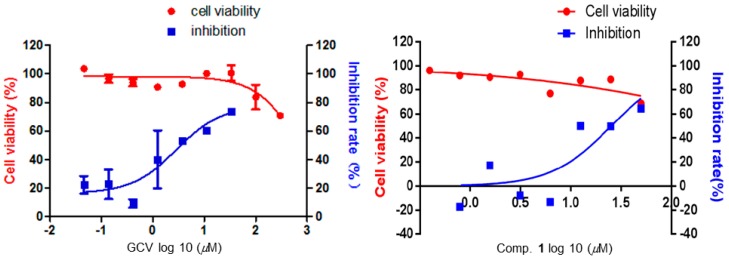
Effects on B95-8 cells viabilities and inhibition on lytic EBV replication of compound **1** was measured using GCV as positive control in vitro. B95-8 cells (5 × 10^5^/well) were cultivated with designated concentrations of compounds in present of 12-*O*-tetradecanoyl-phorbol-13-acetate (TPA). The 50% cytotoxic concentration (CC_50_) of **1** was calculated from the dose-response curve by Graphpad5.0 Prism. The 50% effective concentration (EC_50_) value correspond to compound concentrations required to reduce quantitative expression of the copy number of intracellular viral genomic DNA by 50%. Both values of CC_50_ and EC_50_ were obtained as mean values with standard deviations (*n* = 3).

Epstein-Barr virus (EBV, Lymphocryptovirus), a large DNA virus of the *γ*-herpes virus family, preferentially infects human B cells of at least 90% of the worldwide population in a latent state [19]. EBV is generally linked to a group of autoimmune ailments, such as systemic lupus erythematosus [20], multiple sclerosis [21], and rheumatoid arthritis [22]. Currently, anti-EBV drugs like ganciclovir and aciclovir, have efficacy against EBV lytic infections, while the increasing emergence of drug-related toxicity, cross-resistance, and side effects also limit their clinical application [23,24,25]. As a successive biochemical research on this herb, compound **1** was carried out an inhibition assay on lytic DNA replication of EBV in B95-8 cells in terms of our previous procedure [16]. Comparing the results with the reported compounds (hyperjaponols A–G), **1** exhibited a moderate effect with EC_50_ 25.00 μM, and the value of a CC_50_ higher than 50 µM (Figure 4 and Table 2).

## 3. Materials and Methods

### 3.1. General Experiments

Optical rotation was recorded on a JASCO P-2200 digital polarimeter (JASCO, Tokyo, Japan). IR, UV, and ECD spectra were measured by Bruker Vertex 70 (Brucker Co., Karlsruhe, Germany), Varian Cary 50 (Varian Medical Systems, Salt Lake City, UT, USA), and JASCO J-1700 (JASCO, Tokyo, Japan) apparatuses, respectively. HRESIMS was carried out on Agilent 6530 Accurate-Mass Q-TOF LC/MS spectrometer (Agilent Technologies, California, CA, USA) employed with positive ion mode with nebulizer pressure at 2.0 bar, dry gas temperature at 593 K, and assembled with Agilent Extend-C_18_ column (50 mm × 2.1 mm, 1.8 μm) under the mobile phase (MeOH/H_2_O, 90/10 (*V*/*V*)) with a flow rate of 0.5 mL/min. NMR spectra were run on a Bruker AM-600 spectrometer (Brucker Co., Karlsruhe, Germany), ^1^H-NMR (600 MHz), ^13^C-NMR (150 MHz), using TMS as the internal standard. Chemical shifts of ^1^H and ^13^C-NMR were reported in ppm relative to the solvent peaks of CDCl_3_ (δ_H_ 7.24 ppm; δ_C_ 77.23 ppm). DEPT 135, HSQC (acquired size 512, 256; spectral size 1024, 1024), HMBC (acquired size 1024, 128; spectral size 2048, 1024), ^1^H-^1^H COSY (acquired size 1024, 128; spectral size 1024, 1024), NOESY (acquired size 1024, 128; spectral size 1024, 1024) experiments were performed. Silica gel (0.12–0.2 and 0.2–0.3 mm, Yantai Chemical Co. Ltd., Yantai, China), MCI gel (Mitsubishi Chemical Co., Tokyo, Japan), ODS (YMC Co., Tokyo, Japan), and Sephadex LH-20 (Mitsubishi Chemical Co., Tokyo, Japan) were used for column chromatography. Thin-layer chromatography (GF 254, Yantai Chemical Co. Ltd., Yantai, China) was performed for monitoring isolates under an ultraviolet-visible detector with λ 254 nm. Semi-preparative high performance liquid chromatography (HPLC) was carried out by a LC 3050 analysis of HPLC system (CXTH, Beijing, China) with a RP-C_18_ column (5 μm, 10 × 250 mm, Welchrom^®^, Shanghai, China).

### 3.2. Plant Material

The aerial parts of herbs (*H. japonicum*) were collected from Da-Bie Mountain area, Qichun County, Hubei Province, P. R. China, in October 2016, and were authenticated by Professor Jianping Wang, Huazhong University of Science and Technology. A voucher specimen (no. 2016-1011) was deposited at the Herbarium of Hubei Key Laboratory of Biotechnology of Chinese Traditional Medicine, School of Life Science, Hubei University, Wuhan, P. R. China.

### 3.3. Extraction and Isolation

The air-dried aerial parts of herbs (*H. japonicum*) (4 kg) were percolated with 95% aqueous EtOH (10 L) at 40 °C for 72 h to produce a crude extract (300 g), which was subjected to silica gel column (silica gel, 0.12–0.2 mm, 1.5 kg; column, 10 × 75 cm) chromatography (silica gel CC) eluted successively with the gradient mobile phases of petroleum ether (12 L), chloroform (8 L), and ethyl acetate (8 L). The petroleum ether fraction was subjected to silica gel column chromatography (petroleum ether/acetone, 100:1 to 5:1) to produce seven fractions (Fractions 1–7). With the aid of TLC analyses, fraction 3 was chosen and subjected on silica gel column (petroleum ether/acetone, 50:1 to 5:1) to yield four subfractions (fractions 3.1–3.4). Fraction 3.2 was purified using an ODS column with a gradient elution (MeOH–H_2_O (50:50 to 100:0), to produce four subfractions (fractions 3.2.1–3.2.4). Fraction 3.2.3 was further repurified by semi-preparative HPLC (MeOH–H_2_O, 85:15; flow rate, 2.0 mL/min; t*_R_*, 37.5 min) to afford **1** (2.1 mg).

Hyperjaponol H (**1**): white amorphous powder; [α]${}_{D}^{20}$ +16.4 (*c* 0.06, CHCl_3_); UV (CH_3_OH) λ_max_ (log ε) 243 (3.71) (Supplementary Materials Figure S8), 329 (3.90) nm; IR (KBr) *ν*_max_ 3455, 2966, 2932, 2874, 1654, 1612, 1523, 1464 cm^−1^; ECD λ_max_ (∆ε) 231 (+10.34), 352 (−0.79) nm; ^1^H and ^13^C-NMR data, see Table 1; HRESIMS: *m*/*z* 459.3119 [M + H]^+^ (calcd for C_28_H_43_O_5_ 459.3110) (Supplementary Materials Figure S1).

### 3.4. Anti-EBV Assay

Regarding the pathogenicity of EBV infection, viral replication plays a critical role, and the inhibition of viral replication is a crucial parameter used to assess anti-virus activity of drugs. Hence the inhibitory activity on EBV DNA replication of compound **1** was investigated using previous procedures [26,27,28,29]. The cytotoxicity of compound **1** towards B95-8 cells was assessed by the AlamarBlue^®^ cell viability assay (Thermo Fisher Scientific, Waltham, MA, USA) according to the manufacturer′s protocol. Thereupon, the antiviral activity of compound 1 against the lytic replication of EBV in B95-8 cells was measured using a qPCR assay to assess the intracellular viral DNA copy number, an accurate and rapid assessment of the efficacy of EBV DNA inhibitors as reported [26]. Extraction of the EBV genomic DNA, determination of the viral DNA copy number, and evaluation of the intracellular viral genomic DNA were undertaken referring to our previously described method [16].

## 4. Conclusions

Hyperjaponols H (**1**), a new filicinic acid-based meroterpenoid, with a 6/6/10 ring system *trans*-fused by hetero-Diels–Alder cycloaddition between a germacrane sesquiterpenoid and a filicinic acid moiety, was discovered from *Hypericum japonicum*. The structure and absolute stereocenters were attributed to the analyses of extensive spectroscopic data and the Cotton effect of ECD undergoing a comparison with previously reported ones. Primary bioactivity screening suggested that **1** had a moderate inhibitory effect on lytic EBV DNA replication with the EC_50_ value of 25.00 μm.

## Figures and Tables

**Table 1:** ^1^H-NMR (600 MHz) and ^13^C-NMR (150 MHz) spectral data of compound **1** in CDCl_3_ (δ in ppm, *J* in Hz).

Position	δ_H_ (*J*)	δ_C_	Position	δ_H_ (*J*)	δ_C_
1		84.9	14	1.00 *d* (7.0)	16.4
2	1.47 m	36.1	15	1.02 s	21.3
3	0.91 m	25.2	1′		188.7
	1.32 m		2′		104.6
4	1.58 m	31.4	3′		197.1
	1.51 m		4′		48.5
5	2.65 m	33.9	5′		173.1
6	5.59 *dd* (16.2, 4.7)	136.8	6′		101.9
7	5.47 *d* (16.2, 8.0)	127.5	7′	2.74 *dd* (16.6, 5.2)	21.8
8	2.31 t (7.0)	50.0		1.69 *dd* (16.6, 11.3)	
9	1.90 m	24.7	8′		207.9
	1.43 m		9′	3.93 sept (13.5, 6.7)	35.5
10	1.79 *ddd* (14.7, 10.0, 4.5)	32.1	10′	1.10 *d* (6.7)	19.15
	2.03 dt (14.8, 3.9)		11′	1.11 *d* (6.7)	19.24
11		73.0	12′	1.24 s	24.1
12	1.19 s	27.9	13′	1.29 s	25.4
13	1.20 s	28.7			

**Table 2:** Anti-EBV activities of positive control ganciclovir (GCV), **1**, and the reported compounds (hyperjaponols **A**–**G**) (*µ*M).

Compounds	CC_50_^a^	EC_50_^b^	Selectivity Index (CC_50_/EC_50_)
GCV	>300	2.86	>104.50
**1**	>50	25.00	>2
(+)-hyperjaponol **A**	>41.35	10.33	>4.00
(−)-hyperjaponol **A**	>300	119.4	>2.50
(+)-hyperjaponol **B**	>30	0.57	>52.63
(−)-hyperjaponol **B**	>120	6.60	>18.18
(+)-hyperjaponol **C**	31.75	−	−
(−)-hyperjaponol **C**	17.78	−	−
hyperjaponol **D**	48.05	0.49	106.78
hyperjaponol **E**	60.49	17.53	3.45
hyperjaponol **F**	41.62	14.47	2.87
hyperjaponol **G**	>300	>300	−

^a^: 50% cytotoxic concentration; ^b^: 50% effective concentration.

## Supplementary Materials

HRESIMS, NMR, UV, and IR spectra of compound **1** are available online.
