# Supplementary material for: Hyperjaponol H, A New Bioactive Filicinic Acid-Based Meroterpenoid from Hypericum japonicum Thunb. ex Murray
Source: Molecules. 2018 Mar 18;23(3):683. doi: 10.3390/molecules23030683 (PMC6017031; doi:10.3390/molecules23030683)
Supplement: Supplementary file 1 [file molecules-23-00683-s001.pdf]

Hyperjaponol H, A New Bioactive Filicinic Acid-based Meroterpenoid from *Hypericum japonicum* Thunb. ex Murray

Rongrong Wu [1,†], Zijun Le [2,†], Zhenzhen Wang [3], Shuying Tian [1], Yongbo Xue [3], Yong Chen [1], Linzhen Hu [1, *] and Yonghui Zhang [3]

[1] National & Local Joint Engineering Research Center of High-throughput Drug Screening Technology, Hubei Key Laboratory of Biotechnology of Chinese Traditional Medicine, School of Life Science, Hubei University, Wuhan 430062, Hubei Province, P. R. China; E-mails: wrr08291798@163.com (R. R. W.); TSY15008202259@163.com (S. Y. T.); yongchen101610@163.com (Y. C.)

[2] Wuhan Rayson School, Wuhan 430040, Hubei Province, P. R. China; E-mail: zijunle2018@163.com (Z. J. L.)

[3] Hubei Key Laboratory of Natural Medicinal Chemistry and Resource Evaluation, School of Pharmacy, Tongji Medical College, Huazhong University of Science and Technology; Wuhan 430030, Hubei, China; E-mails : wzz75283@163.com (Z. Z. W.); yongboxue@hust.edu.cn (Y. B. X.); zhangyh@mails.tjmu.edu.cn (Y. H. Z.)

† These authors contributed equally to this work.

* Author to whom correspondence should be addressed; E-mail: linzhenhu@hubu.edu.cn (L. Z. H.); Tel.: +86-27-88668023 (L. Z. H.)

Content

Figure S1. HRESIMS spectrum of hyperjaponol H (**1**) 1

Figure S2. ^{1}H NMR spectrum of hyperjaponol H (**1**, in $CDCl_3$) 2

Figure S3. ^{13}C NMR and DEPT135 spectra of hyperjaponol H (**1**, in $CDCl_3$) 3

Figure S4. HSQC spectrum of hyperjaponol H (**1**, in $CDCl_3$) 4

Figure S5. HMBC spectrum of hyperjaponol H (**1**, in $CDCl_3$) 5

Figure S6. ^{1}H–^{1}H COSY spectrum of hyperjaponol H (**1**, in $CDCl_3$) 6

Figure S7. NOESY spectrum of hyperjaponol H (**1**, in $CDCl_3$) 7

Figure S8. UV spectrum of hyperjaponol H (1, in methanol) 8

Figure S9. IR spectrum of hyperjaponol H (**1**, KBr disc) 9

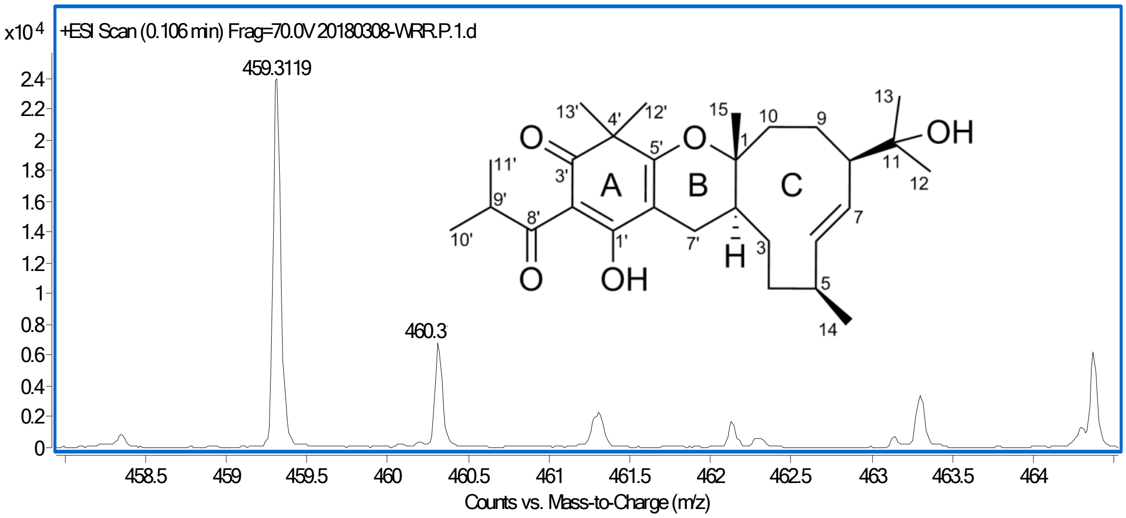

Figure S1. HRESIMS spectrum of hyperjaponol H (**1**)

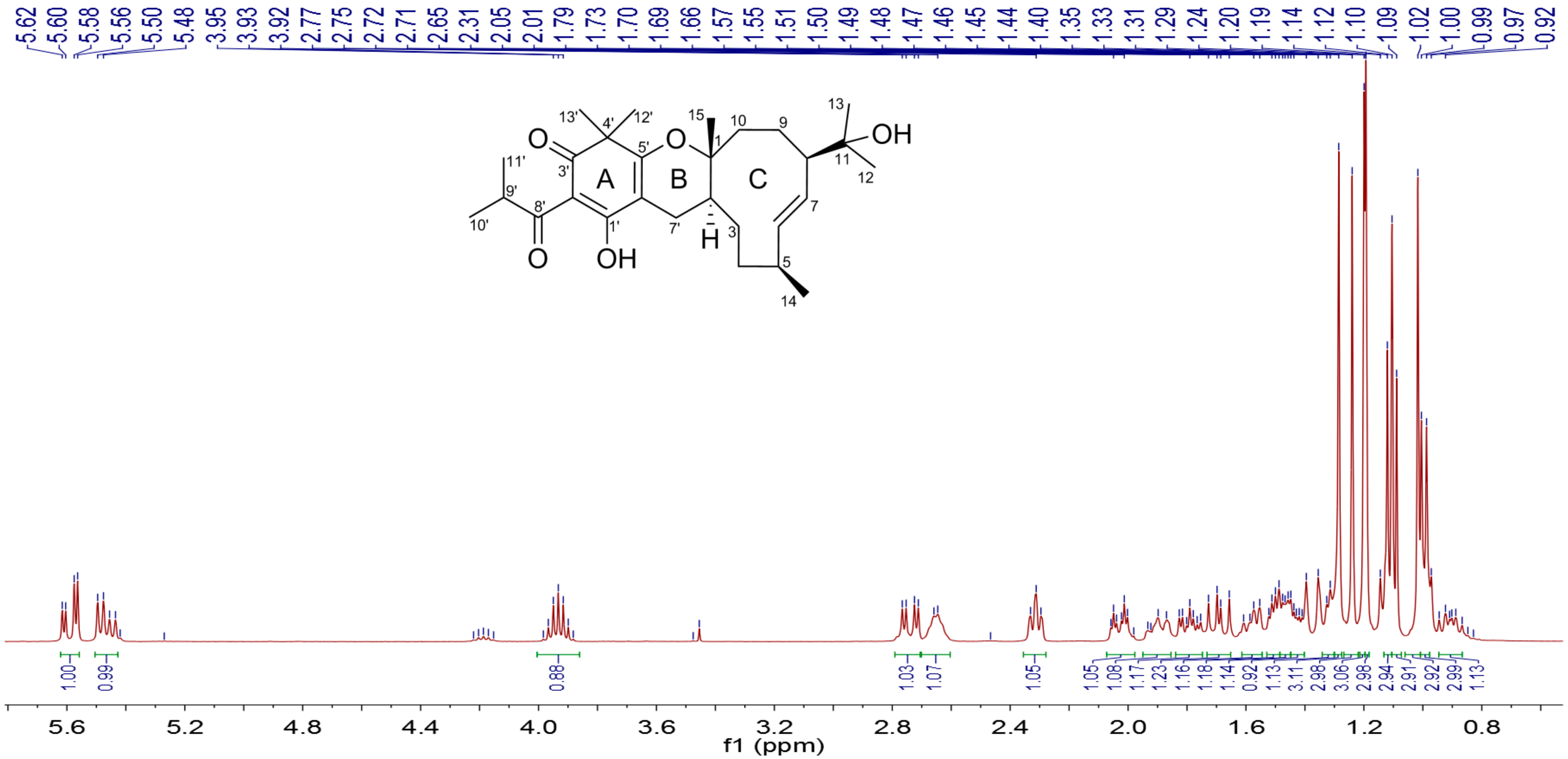

Figure S2. ^{1}H NMR spectrum of hyperjaponol H (**1**, in $CDCl_3$)

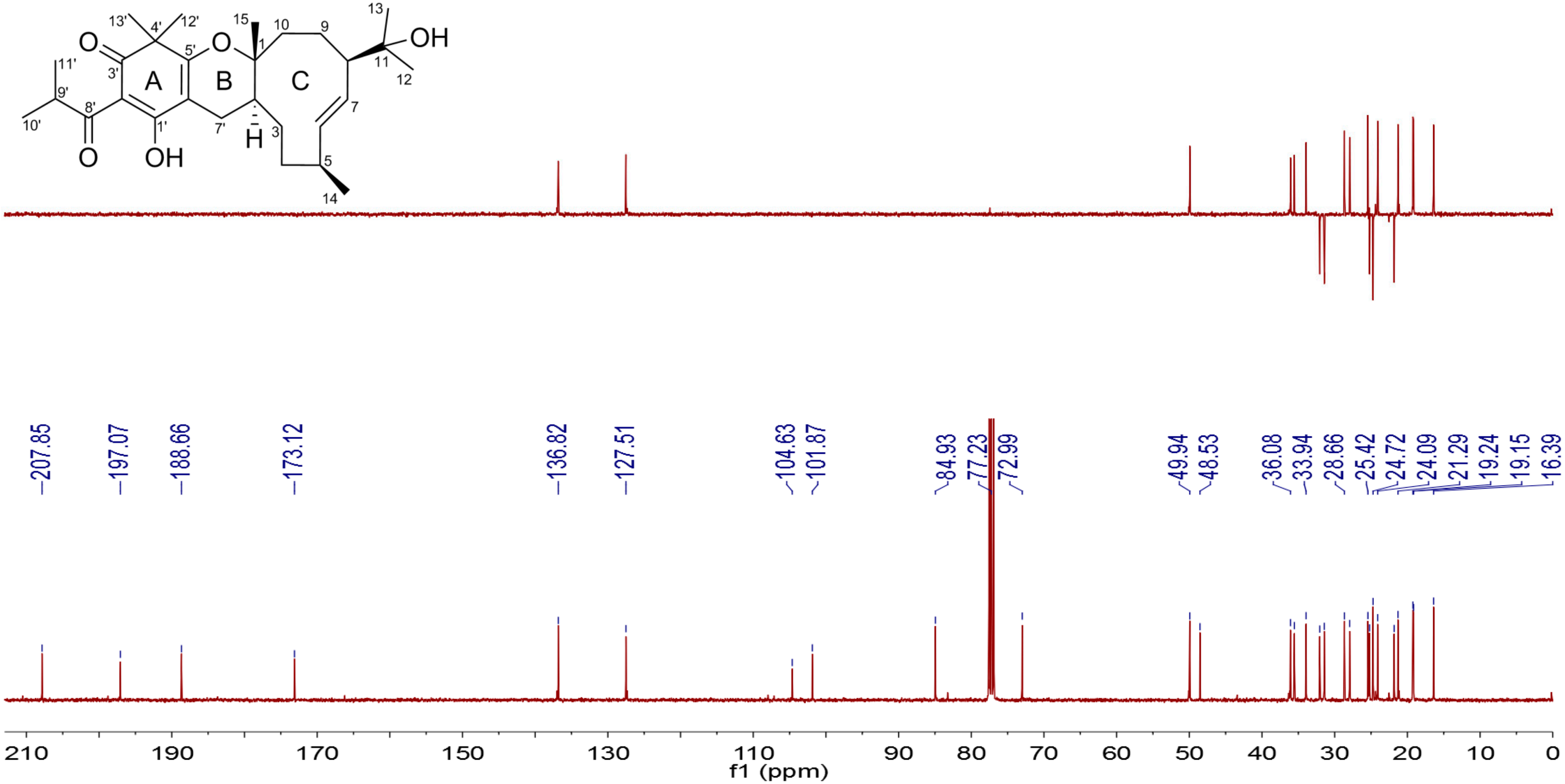

Figure S3. ^{13}C NMR and DEPT135 spectra of hyperjaponol H (**1**, in $CDCl_3$)

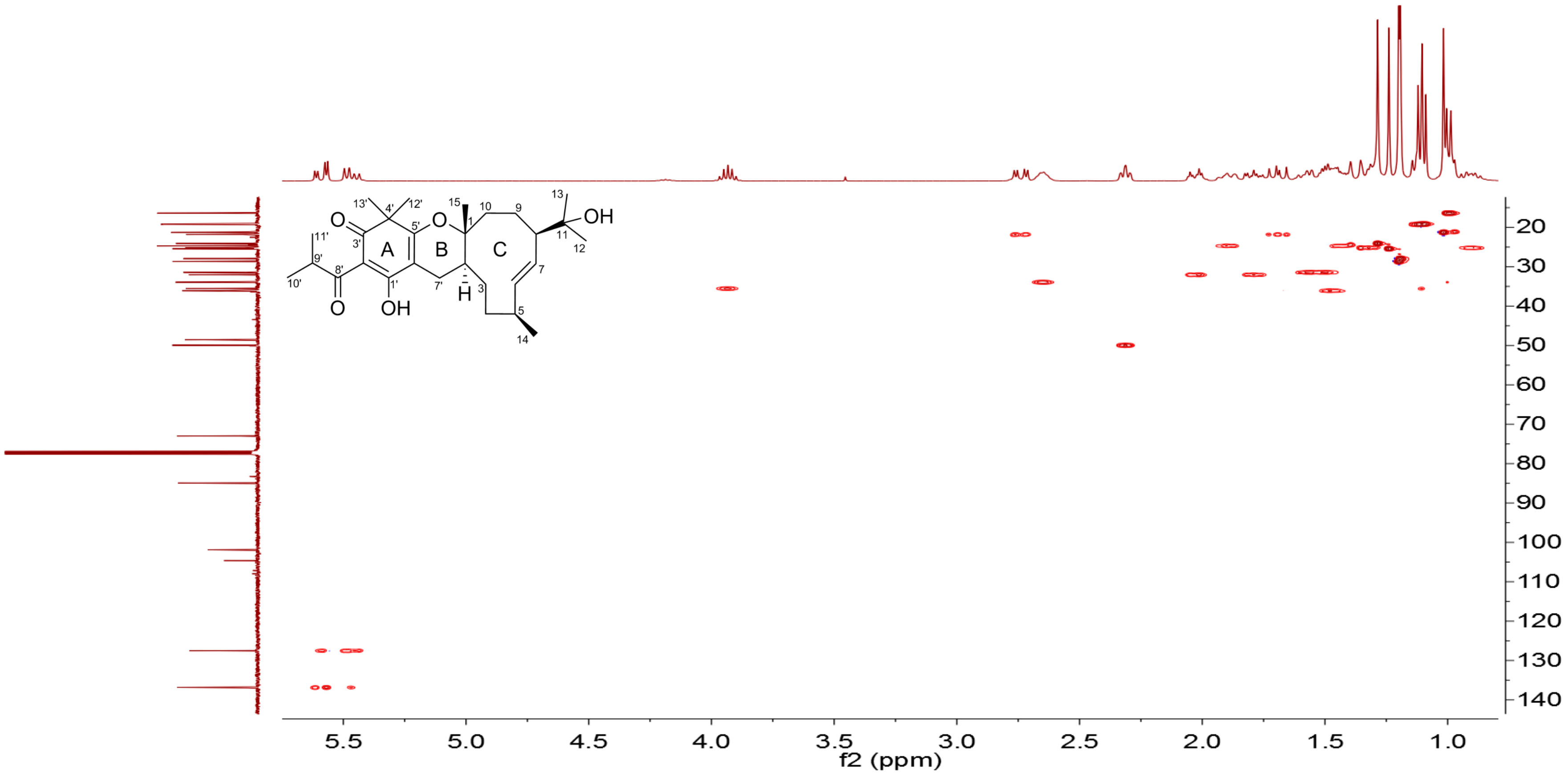

Figure S4. HSQC spectrum of hyperjaponol H (**1**, in $CDCl_3$)

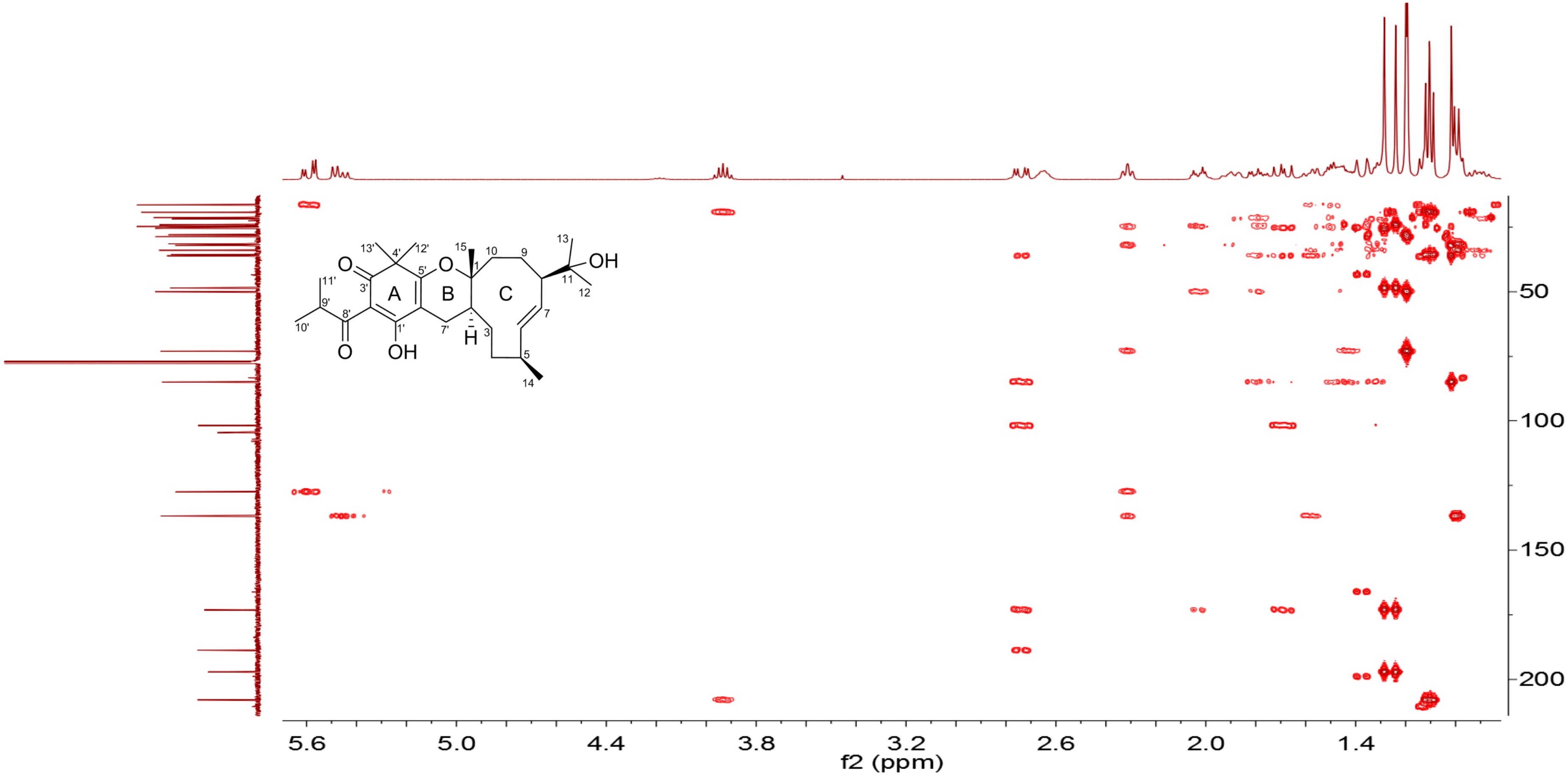

Figure S5. HMBC spectrum of hyperjaponol H (**1**, in $CDCl_3$)

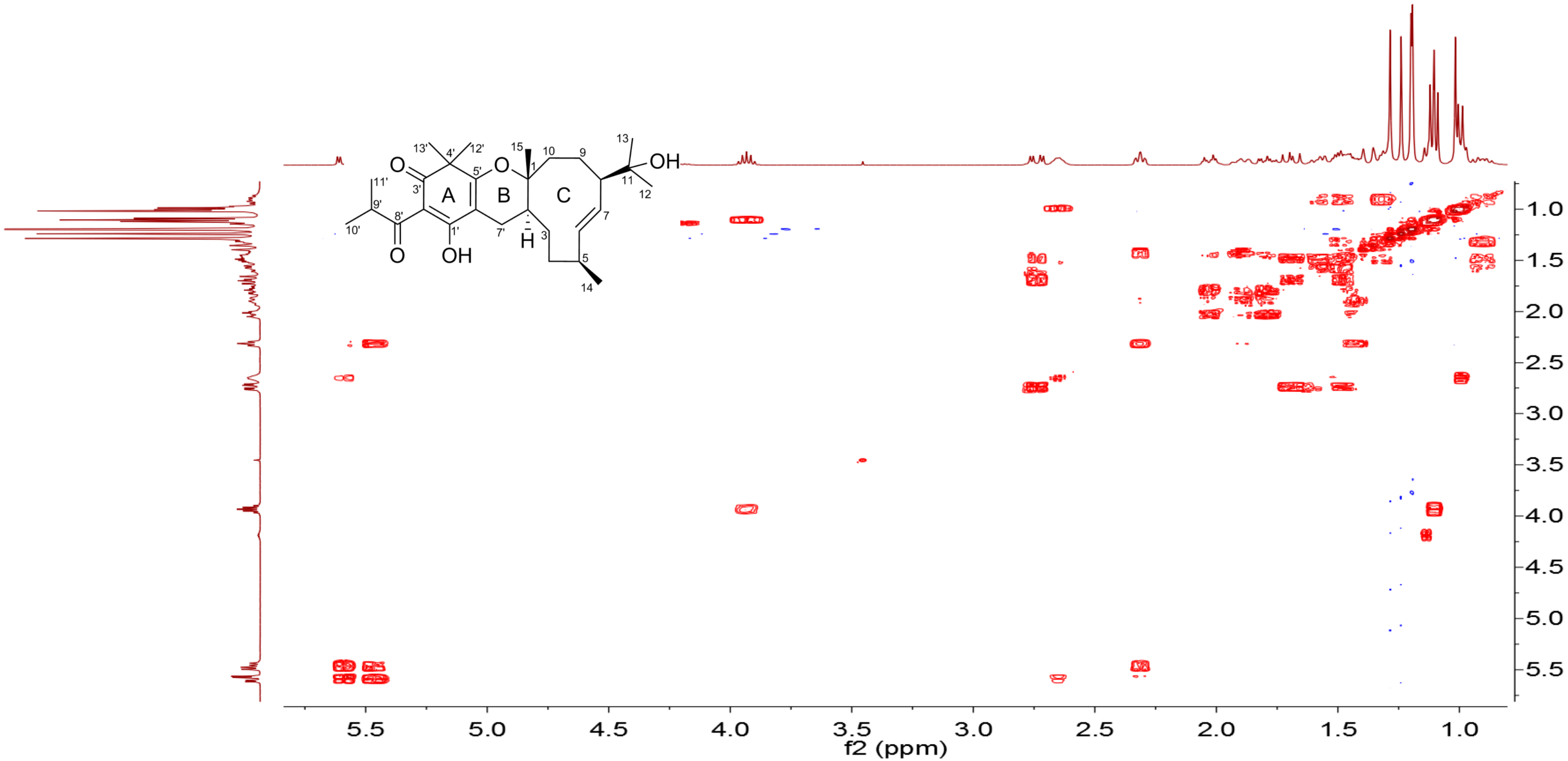

Figure S6. ^{1}H–^{1}H COSY spectrum of hyperjaponol H (**1**, in $CDCl_3$)

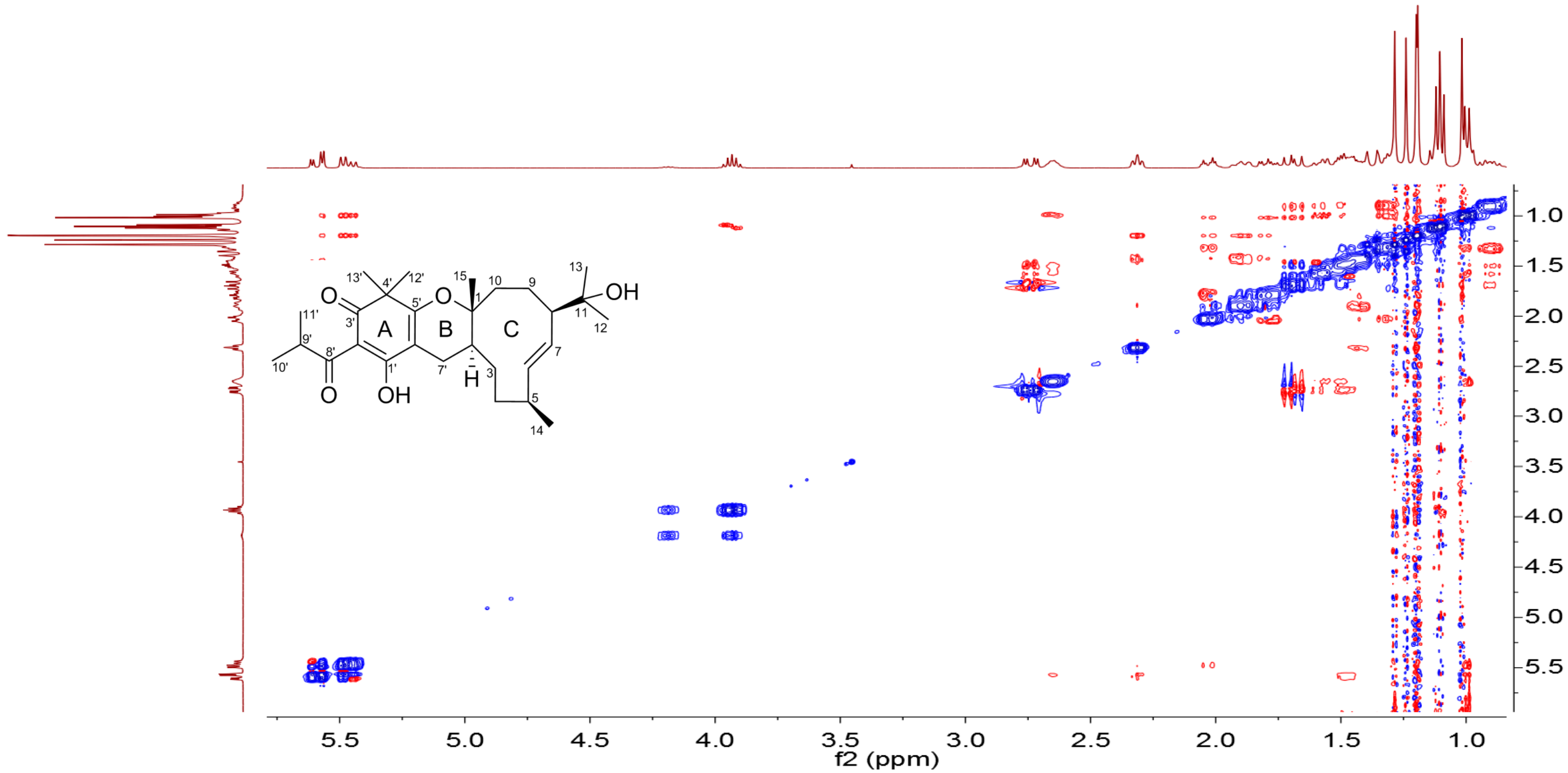

Figure S7. NOESY spectrum of hyperjaponol H (**1**, in $CDCl_3$)

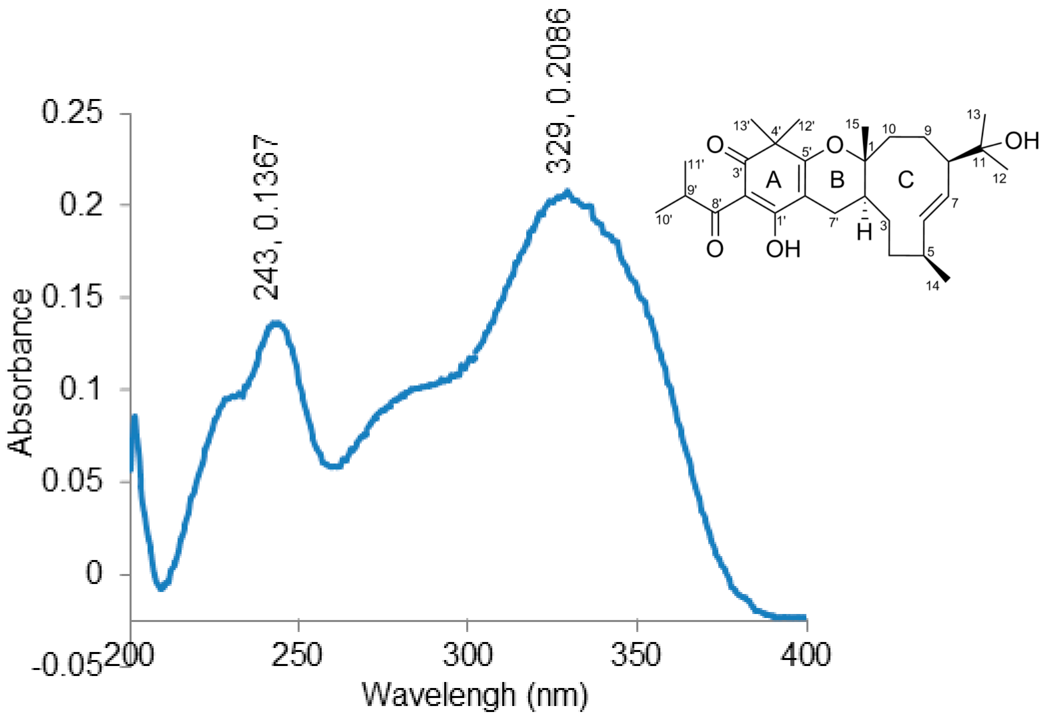

Figure S8. UV spectrum of hyperjaponol H (**1**, in methanol)

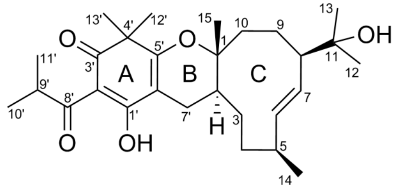

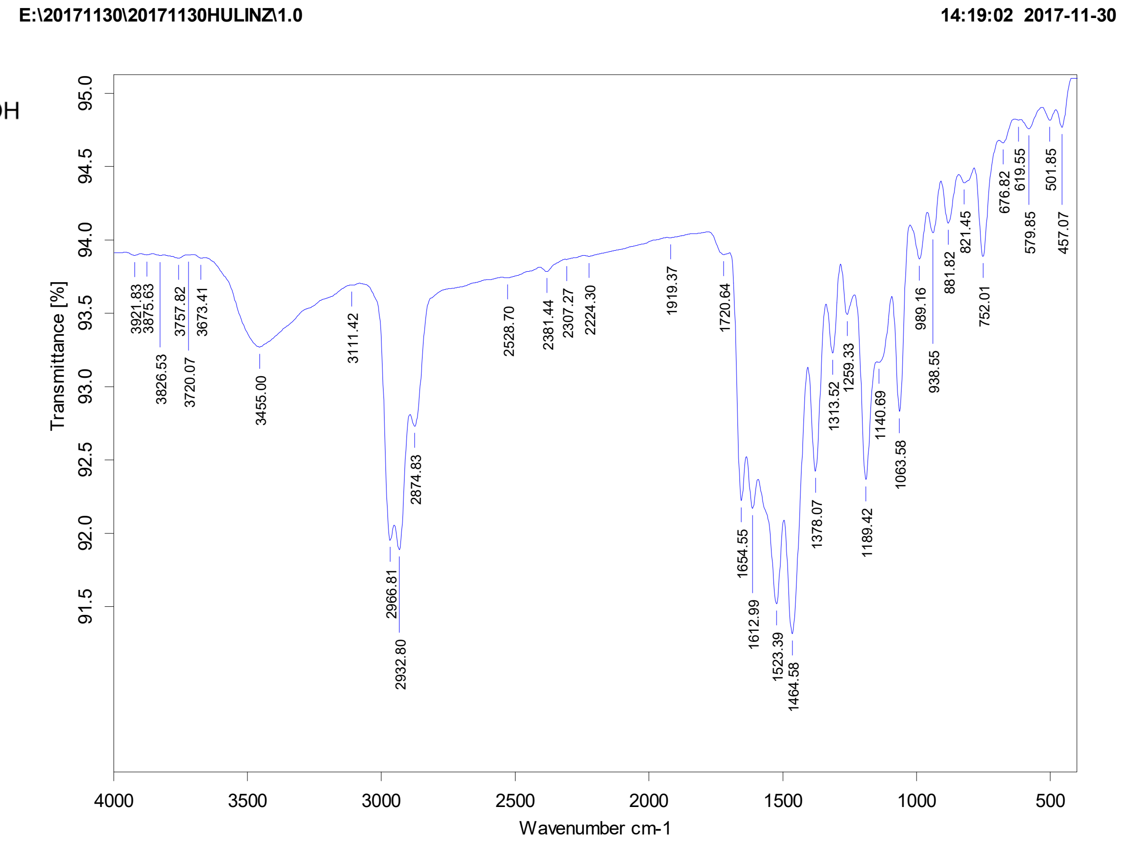

Figure S9. IR spectrum of hyperjaponol H (**1**, KBr disc)
